# Случай клинически агрессивного течения первичного гиперпаратиреоза, алгоритм дифференциальной диагностики

**DOI:** 10.14341/probl13159

**Published:** 2022-12-20

**Authors:** А. С. Матюшкина, А. М. Горбачева, А. В. Ткачук, А. К. Еремкина, Н. Г. Мокрышева

**Affiliations:** Национальный медицинский исследовательский центр эндокринологии; Национальный медицинский исследовательский центр эндокринологии; Национальный медицинский исследовательский центр эндокринологии; Национальный медицинский исследовательский центр эндокринологии; Национальный медицинский исследовательский центр эндокринологии

**Keywords:** первичный гиперпаратиреоз, синдром гиперпаратиреоза с опухолью челюсти, рак околощитовидной железы, аденома околощитовидной железы, «бурая» опухоль

## Abstract

Первичный гиперпаратиреоз (ПГПТ) — значимое эндокринное заболевание, обусловленное повышением продукции паратиреоидного гормона (ПТГ) измененными околощитовидными железами (ОЩЖ) и нарушением механизмов регуляции сывороточных концентраций кальция. Эти изменения могут привести к нефролитиазу, остеопорозу, эрозивно-язвенному поражению желудочно-кишечного тракта, ряду менее специфичных симптомов (тошноте, рвоте, слабости, усталости и прочему). Этиологически более чем в 85% случаев ПГПТ является следствием спорадической солитарной аденомы или гиперплазии ОЩЖ, однако в 1–3% случаев причиной становится карцинома ОЩЖ, в том числе в составе различных наследственных синдромов. Отмечена важность своевременного обследования на предмет ПГПТ пациентов с характерными клиническими проявлениями данного заболевания и — при агрессивном течении — настороженности в отношении карцином ОЩЖ. В то же время тяжесть клинической картины и даже наличие подозрительных признаков, характерных для наследственных форм карцином ОЩЖ, не всегда являются следствием злокачественного процесса. Мы представляем описание молодой пациентки с тяжелым течением ПГПТ, множественными переломами и объемным образованием верхней челюсти, развившимися вследствие типической аденомы ОЩЖ. Дополнительно освещен алгоритм пред- и послеоперационной дифференциальной диагностики для таких больных.

## Abstract (en)

Primary hyperparathyroidism (PHPT) is a significant endocrine disease caused by increased production of parathyroid hormone (PTH) by altered parathyroid glands and violation of the mechanisms of regulation of serum calcium concentrations. These changes can lead to nephrolithiasis, osteoporosis, erosive and ulcerative lesions of the gastrointestinal tract, a number of less specific symptoms (nausea, vomiting, weakness, fatigue, etc.). Etiologically, in more than 85% of cases, PHPT is a consequence of sporadic solitary adenoma or hyperplasia parathyroid glands, however, in 1–3% of cases, the cause is carcinoma of parathyroid glands , including as part of various genetic syndromes. The importance of timely examination for PHPT of patients with characteristic clinical manifestations of this disease and — with an aggressive course — alertness towards carcinomas of parathyroid glands was noted. At the same time, the severity of the clinical picture and even the presence of suspicious signs characteristic of hereditary forms of carcinomas of parathyroid glands are not always a consequence of the malignant process. We present a description of a young patient with a severe course of PHPT, multiple fractures and a voluminous tumor of the upper jaw, developed as a result of a typical adenoma of parathyroid glands. Additionally, the algorithm of pre- and postoperative differential diagnosis for such patients is highlighted.

## АКТУАЛЬНОСТЬ

Первичный гиперпаратиреоз (ПГПТ) — эндокринное заболевание, обусловленное повышением продукции паратиреоидного гормона (ПТГ) измененными околощитовидными железами (ОЩЖ) [1] и нарушением механизмов регуляции сывороточных концентраций кальция. Эти изменения могут привести к нефролитиазу, остеопорозу, эрозивно-язвенному поражению желудочно-кишечного тракта, ряду менее специфичных симптомов (тошноте, рвоте, слабости, усталости и проч.) [2]. При активном скрининге ПГПТ частота симптомных форм не превышает 20%, все остальные случаи протекают без яркой клинической манифестации [3][4]. В то же время, по данным анализа Российского регистра ПГПТ (n=1914), частота манифестных форм вРоссийской Федерации достигает 67% [5]. Это связано преимущественно с отсутствием программ скрининга и поздней диагностикой данного заболевания.

Этиологически более чем в 85% случаев ПГПТ обусловлен спорадической солитарной аденомой или гиперплазией ОЩЖ, однако в 1–3% случаев причиной является карцинома ОЩЖ, в частности, в составе различных наследственных синдромов. Считается, что для карцином характерно более тяжелое течение: выраженная гиперкальциемия (уровень общего кальция более 3,0 ммоль/л), более чем троекратное превышение концентрации ПТГ относительно верхней границы референсного диапазона и значительные размеры образования (более 3 см) [6][7].

Вышеизложенное обусловливает важность своевременного обследования пациентов с характерными клиническими проявлениями ПГПТ и — при агрессивном течении — настороженности в отношении карцином ОЩЖ. В то же время тяжесть клинической картины и даже наличие подозрительных признаков, характерных для наследственных форм карцином ОЩЖ, не всегда являются следствием злокачественного процесса.

Мы представляем описание молодой пациентки с тяжелым течением ПГПТ, множественными переломами и объемным образованием верхней челюсти, развившимися вследствие аденомы ОЩЖ. В статье освещен подробный алгоритм пред- и послеоперационной дифференциальной диагностики для таких больных.

## Описание клинического случая

Пациентка С., 39 лет, поступила в отделение патологии околощитовидных желез и нарушений минерального обмена ФГБУ «НМИЦ эндокринологии» Минздрава России в декабре 2021 г. с жалобами на боли в костях, онемение в конечностях, ощущения перебоев в работе сердца.

## Anamnesis morbi

Считает себя больной с 2016 г., когда при стационарном обследовании по поводу острой боли в поясничной области была диагностирована мочекаменная болезнь. Со слов, в тот период пациентка отметила самостоятельное отхождение более 12 конкрементов; в дальнейшем не обследовалась, лечение не получала. Также в течение более 14 лет пациентка отмечала снижение концентрации гемоглобина крови (в том числе до значений менее 60 г/л) без очевидных источников кровопотерь и при наличии в рационе продуктов, богатых железом.

Значительное ухудшение самочувствия наблюдалось с февраля 2021 г., когда при падении с высоты собственного роста (поскользнулась на льду) пациентка перенесла перелом правой ключицы. По месту жительства был выполнен металлоостеосинтез, с тех пор появились эпизоды головокружения, слабости, тошноты, рвоты, потери памяти.

Тогда же, в феврале 2021 г., пациентка отметила появление объемного образования верхней челюсти слева. В период c апреля по май находилась на лечении в стоматологическом отделении по месту жительства с диагнозом «Гигантоклеточный эпулис верхней челюсти в области 23, 24, 27, 28 зубов с кровотечением». При компьютерной томографии (КТ) лицевого скелета от 22.04.2021 г. выявлялись воспалительные изменения альвеолярного отростка верхней челюсти слева с наличием жидкостного скопления (вероятно, абсцесса). Тогда же проведено удаление новообразования челюсти. По данным гистологического исследования строение удаленной опухоли соответствовало гигантоклеточной опухоли (остеобластокластоме).

Через несколько месяцев пациентка была госпитализирована с гемартрозом коленных суставов (медицинская документация не предоставлена), а через несколько дней после выписки оступилась и упала. При обследовании выявлен разрыв четырехглавой мышцы правого бедра, выполнена иммобилизация правой нижней конечности. С тех пор пациентка передвигалась на костылях или при помощи кресла-каталки.

Учитывая данные анамнеза, было заподозрено наличие гемопролиферативного заболевания, в связи с чем в апреле 2021 г. выполнена стернальная пункция. По результатам исследования плазматические клетки не определялись. Впоследствии, в июне 2021 г., была госпитализирована в гематологическое отделение по месту жительства. В стационарных условиях впервые проведено ультразвуковое исследование (УЗИ) щитовидной железы (ЩЖ), в ходе которого было визуализировано образование, деформирующее нижний полюс ЩЖ, пониженной эхогенности, округло-овальной формы, неоднородной структуры, с ровными четкими контурами, размерами 24×14×15 мм. Сцинтиграфия подтвердила локализацию образования.

Чуть позже, при попытке встать на костыли, наступила на левую ногу, после чего почувствовала боль и хруст в левой голени. При обследовании был диагностирован закрытый патологический перелом диафиза левой большеберцовой кости без смещения. Впервые была проведена оценка показателей минерального обмена, диагностирован первичный гиперпаратиреоз (кальций общий 4,87–4,88 ммоль/л, кальций ионизированный 2,1 ммоль/л при референсных значениях лаборатории 2,15–2,55, повышение сывороточной концентрации ПТГ до 767,3 пг/мл (16–46)).

В рамках скрининга осложнений ПГПТ обращало на себя внимание значимое повышение концентрации щелочной фосфатазы — до 3473 Ед/л (40–150). Также у пациентки имелась анемия со снижением концентрации гемоглобина до 90 г/л (железо 9,6 мкмоль/л, общая железосвязывающая способность сыворотки 40,86 мкмоль/л, трансферрин 2,8 г/л, ферритин 155 мкг/л, насыщение трансферрина железом 23,49%). По данным трепанобиопсии от 24.06.2021 г. диагностирован резко выраженный миелофиброз с очагами остеокластической резорбции кости, признаков опухолевого роста не выявлено. В обеих почках при УЗИ визуализировались конкременты до 11 мм, отмечалось снижение скорости клубочковой фильтрации (СКФ) до 40,5 мл/мин/1,73 м2. При эзофагогастродуоденоскопии данных за эрозивно-язвенное поражение желудочно-кишечного тракта не получено.

В июле 2021 г. пациентке по месту жительства была проведена селективная левосторонняя паратиреоидэктомия, гистологическое заключение пациентка не представила В послеоперационном периоде назначалась терапия: холекальциферолом 7000 МЕ/сут, альфакальцидолом 1 мкг/сут, карбонатом кальция 500 мг 2 р/сут. Впоследствии карбонат кальция был отменен.

Динамика лабораторных показателей представлена в таблице 1.

При поступлении пациентка получала альфакальцидол 1 мкг в сутки, холекальциферол 7000 МЕ 1 раз в сутки (указанная дозировка — более 2 мес), железа (III) гидроксид полимальтозат 100 мг 1 раз в сутки.

**Table:** Таблица 1. Динамика лабораторных показателей пациентки С.Table 1. Dynamics of laboratory parameters of patient S. Примечание. ПТГ — паратгормон; 25(ОН)D — 25-гидроксикальциферол; Са ион. — ионизированный кальций; Са скорр. — кальций, скорректированный на альбумин; ЩФ — щелочная фосфатаза; рСКФ — расчетная скорость клубочковой фильтрации.

Дата	25 (ОН)D, нг/мл	ПТГ, пг/мл (16–46)	Са ион., ммоль/л (1,15–1,29)	Са скорр., ммоль/л (2,15–2,55)	Калий, ммоль/л (3,5–5,8)	ЩФ, Ед/л (98–279)	Фосфор, ммоль/л (0,87–1,45)	Креатинин, мкмоль/л(44–80)	рСКФ, мл/мин/1,73 м2
22.07.2021	20	83,3	1,18						
23.07.2021				2,25	6,6	2611,4	1		
11.08.2021					5,1			126	46
20.09.2021	11,3	261,6	1,24	2,25		982,6	1,05	112	53

## Anamnesis vitae

Из сопутствующих заболеваний, по данным медицинской документации, у пациентки имелись поверхностный гастрит, гиперпластические полипы желудка. Наследственность не отягощена. Менструальный цикл нерегулярный с начала 2021 г., до этого — регулярный, в анамнезе — две беременности и двое родов.

## Результаты физикального, лабораторного и инструментального исследования

При поступлении отмечалось повышение сывороточной концентрации ПТГ до 738,8 пг/мл [ 15–65] при умеренной гипокальциемии (Са скорр. 2,11 ммоль/л [ 2,15–2,55]), нормофосфатемии (1,25 ммоль/л [ 0,74–1,52]), гипокальциурии (0,8 ммоль/сут [ 2,5–8]). В отделении был инициирован прием карбоната кальция, проведена титрация дозы альфакальцидола до 3 мкг в сутки. На фоне терапии отмечена положительная динамика: концентрация ПТГ снизилась до 322,9 пг/мл, достигнуты низконормальные концентрации кальция крови (Са скорр. 2,15 ммоль/л). При УЗИ ОЩЖ выявлены эхографические признаки образования правой нижней ОЩЖ размерами 1,2×0,8×0,7 см. В отделении проведен скрининг осложнений гиперпаратиреоза, входе которого сохранялось снижение фильтрационной функции почек (рСКФ по CKD-EPI 51 мл/мин/1,73 м2). По данным УЗИ, мультиспиральной компьютерной томографии (МСКТ) подтвержден двусторонний нефролитиаз (диаметром 0,3–0,7 см).

Также обращало на себя внимание значимое повышение сывороточных концентраций маркеров костного обмена: остеокальцин >300 нг/мл [ 11–43], С-концевые телопептиды коллагена I типа 3,61 нг/мл [ 0,3–0,57], щелочная фосфатаза 308 Ед/л [ 40–150]. Впервые проведена рентгенденситометрия, максимальное снижение минеральной плотности кости ниже ожидаемых возрастных значений до -2,8 SD по Z-критерию отмечалось в поясничном отделе позвоночника (L1–L4). Учитывая наличие в анамнезе перелома левой голени, проведена рентгенография: определялся консолидированный перелом в средней трети без смещения (см. рис. 1А). При рентгенографии грудного и поясничного отделов позвоночника данных за наличие компрессионных переломов не получено, однако визуализированы признаки начальной компрессии тел нескольких позвонков (рис. 1 В, С). По данным МСКТ визуализировались признаки фиброзно-кистозного остеита, консолидированных переломов 6 ребра справа и 6–8 ребер слева.

**Figure:**
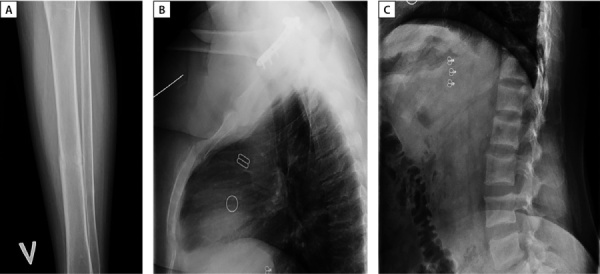
Рисунок 1. А — рентгенография левой голени в прямой проекции. Консолидированный перелом в средней трети без смещения. В — рентгенография грудного отдела позвоночника в боковой проекции. Гиперкифоз, умеренный остеохондроз, спондилез. Начальная компрессия тел Th7, Th8 позвонков по краниальной и каудальной площадкам преимущественно в передней трети позвонков (потеря до 5% массы). С — рентгенография поясничного отдела позвоночника в боковой проекции. Гиперлордоз, остеохондроз, ретролистез L4 и L1 на 2 мм. Начальная компрессия тел L5 (до 8% потери массы по каудальной площадке в задней трети позвонка), L3 ( до 5% в средней трети), L2 ( до 8% в средней трети) позвонков.Figure 1. A — X-ray of the left leg in direct projection. Consolidated fracture in the middle third without displacement. C — X-ray of the thoracic spine in lateral projection. Hyperkyphosis, moderate osteochondrosis, spondylosis. Initial compression of the bodies of Th7, Th8 vertebrae along the cranial and caudal sites, mainly in the anterior third of the vertebrae (loss of up to 5% of the mass). C — radiography of the lumbar spine in lateral projection. Hyperlordosis, osteochondrosis, L4 and L1 retrolisthesis by 2 mm. Initial compression of the bodies of L5 (up to 8% weight loss along the caudal area in the posterior third of the vertebra), L3 (up to 5% in the middle third), L2 (up to 8% in the middle third) of the vertebrae.

В общеклиническом анализе крови отмечалась анемия легкой степени тяжести смешанного генеза (за счет миелофиброза на фоне ПГПТ, В12-дефицитная, ранее также железодефицитная): эритроциты 3,48×1012 кл./л, гемоглобин 108 г/л. Назначена терапия цианокобаламином, фолиевой кислотой, рекомендовано продолжить прием пероральных препаратов железа. С учетом тяжести течения ПГПТ и наличия в анамнезе опухоли челюсти, клинически заподозрен синдром гиперпаратиреоза с опухолью челюсти (HPT-JT).

Для верификации диагноза был выполнен пересмотр гистологических препаратов, по результатам которого верифицирована аденома левой нижней ОЩЖ (рис. 2). Морфологическая картина образования челюсти более всего соответствовала фиброзно-кистозному остеиту с формированием «бурой» опухоли верхней челюсти (рис. 3).

**Figure:**
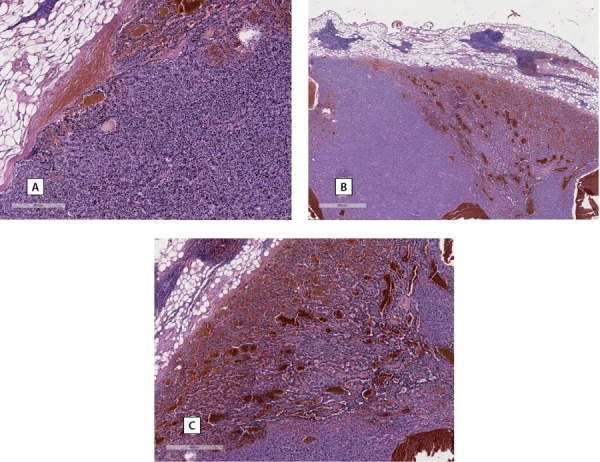
Рисунок 2. А–С — гистологические препараты образования (аденомы) околощитовидной железы.Figure 2. A–C — histological preparations of a formation (adenoma) of the parathyroid gland. Определяется новообразование преимущественно солидного строения с участками фолликулярного и более гнездного строения из небольших клеток с умеренным количеством оптически прозрачной цитоплазмы, округлыми относительно мономорфными темными базофильными ядрами без видимых ядрышек в них. Митотическая активность в 10 исследованных полях зрения (х400) — не определяется. В новообразовании отмечаются фиброзированные прослойки, очаги кровоизлияний. По периферии новообразования присутствует соединительнотканная капсула, за пределами которой в жировой клетчатке отмечаются мелкие фрагменты ткани тимуса, представленные корковым веществом с единичными тельцами Гассаля.

**Figure:**
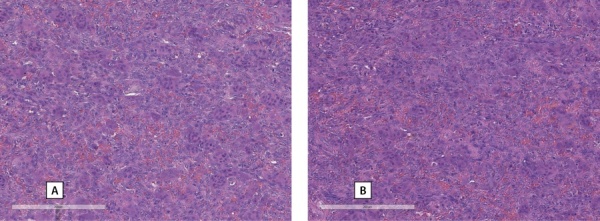
Рисунок 3. А, В — гистологические препараты образования («бурой» опухоли) челюсти.Figure 3. A, B — histological preparations of the formation ("brown" tumor) of the jaw. Определяется остеокластическая резорбция кости с разрастанием фиброзной ткани в межтрабекулярных пространствах с выраженным миелофиброзом. Отмечаются немногочисленные очаги сохранного кроветворения

Кроме того, проведено генетическое исследование панели «гиперпаратиреоз» (методом NGS исследованы гены AIP, AP2S1, CASR, CDC73, CDKN1A, CDKN1B, CDKN1C, CDKN2A, CDKN2C, CDKN2D, DICER1, FAM111A, GATA3, GCM2, GNA11, GNAS, MEN1, POU1F1, PRKAR1A, PRKCA, PTEN, PTTG2, SDHA, SDHB, SDHC, SDHD, TBCE), патогенных мутаций в представленных генах не обнаружено.

## Рекомендации при выписке и дальнейшее наблюдение

Пациентке при выписке была рекомендована медикаментозная терапия препаратами кальция (3000 мг в сутки), витамина D (альфакальцидол 4 мкг в сутки, холекальциферол 2000 МЕ в сутки), а также терапия коррекции анемии смешанного генеза (таблетированные препараты железа, цианокобаламин и фолиевая кислота).

## ОБСУЖДЕНИЕ

Основная сложность ведения данной пациентки заключалась в правильных интерпретации клинических данных и проведении дифференциальной диагностики трех состояний: тяжелого течения ПГПТ вследствие аденомы, рака ОЩЖ и синдрома гиперпаратиреоза с опухолью челюсти (HPT-JT).

Как уже было отмечено выше, частота манифестных форм ПГПТ в Российской Федерации значимо превышает таковую в странах Европы и США [4]. Рак ОЩЖ, в свою очередь, ответственен лишь за 0,1–5% всех случаев ПГПТ, однако эти пациенты отличаются высокой частотой рецидива и неблагоприятным прогнозом при первично не радикальном лечении [8][9].

Сложность заключается в том, что на сегодняшний день отсутствуют способы достоверной предоперационной диагностики карцином ОЩЖ. Чаще всего используется сочетание следующих критериев: выраженная гиперкальциемия (>3 ммоль/л); троекратное или более повышение концентрации ПТГ относительно верхней границы референсного интервала лаборатории и размер образования более 3 см. Также для карцином ОЩЖ характерна манифестация гиперпаратиреоза с наличием тяжелых осложнений, таких как фиброзно-кистозный остеит, рецидивирующий нефролитиаз, гиперкальциемические кризы. В ряде публикаций пальпация объемного образования выступала еще одной клинической детерминантой карциномы: такие образования определялись пальпаторно в 30–76% случаев, тогда как доброкачественные опухоли ОЩЖ обычно не пальпировались [10][11]. К УЗ-характеристикам карцином относят изоэхогенность, неровность контура и неоднородность структуры [12][13]. Однако все описанные критерии обладают достаточно низкой чувствительностью и специфичностью.

ПГПТ является ключевым компонентом синдрома гиперпаратиреоза с опухолью челюсти (HPT-JT) — редкого аутосомно-доминантного заболевания, возникающего при мутации в гене CDC73. Помимо ПГПТ, проявляется это заболевание оссифицирующими фибромами нижней челюсти. Дебют ПГПТ чаще всего происходит в молодом возрасте. При этом частота карцином ОЩЖ в этой когорте больных значительно выше популяционной и достигает 10–21,6%. Точная распространенность синдрома HPT-JT до сих пор неизвестна [14][15].

Важность предоперационной диагностики карцином ОЩЖ, и в частности синдрома HPT-JT, обусловлена различной хирургической тактикой у таких больных. Оптимальный объем операции при ПГПТ в рамках HPT-JT не определен. Возможна паратиреоидэктомия (ПТЭ) при доброкачественном поражении одной ОЩЖ, при поражении нескольких ОЩЖ — субтотальная или тотальная ПТЭ с аутотрансплантацией. При дооперационном подозрении на карциному ОЩЖ — удаление «единым блоком» злокачественной опухоли ОЩЖ, прилежащей доли ЩЖ и перешейка, клетчатки и лимфатических узлов VI зоны на стороне поражения, а также любой спаянной с опухолью мышцы, чтобы предотвратить разрыв капсулы и появление метастазов [16–18].

На этапе первичной диагностики у нашей пациентки обнаруживался целый ряд признаков, характерных для карцином ОЩЖ: ПГПТ с выраженной гиперкальциемией и значительным повышением концентрации ПТГ, наличие тяжелых осложнений, большой размер образования. Сопутствующее образование нижней челюсти и дебют ПГПТ в молодом возрасте (диагноз ПГПТ был установлен в возрасте 39 лет, а первые клинические симптомы возникли в возрасте 34 лет) не позволяли исключить синдром HPT-JT.

При поступлении у пациентки, перенесшей ПТЭ, отмечалось значительное повышение концентрации ПТГ в сочетании с гипокальциемией и гипокальциурией. С учетом исходной тяжести поражения костной ткани это было расценено как проявление синдрома «голодных костей» вследствие повышенной активности остеобластов и поступления кальция в кости [19][20].

Однако, учитывая характер течения заболевания, требовалась морфологическая верификация диагноза, что было выполнено путем пересмотра гистологических препаратов образования ОЩЖ в референс-центре. В итоге диагностирована аденома. Для купирования симптомов гипокальциемии и вторичного гиперпаратиреоза скорректированы дозы препаратов витамина D и кальция с достижением положительной динамики ПТГ при сохранении нормокальциемии.

Важно отметить этап дифференциальной диагностики оссифицирующих фибром челюсти при синдроме HPT-JT и «бурых» опухолей, возникающих при тяжелом течении ПГПТ любой другой этиологии.

Диагноз «бурой» опухоли является в первую очередь клиническим и определяется по наличию при рентгенографии или МСКТ нескольких хорошо выраженных остеолитических очагов на фоне гиперпаратиреоза и гиперкальциемии. Гистологические особенности опухоли являются результатом повышенной активности остеокластов, заменяющих кость реактивной волокнистой тканью, вследствие чего неспецифичны и могут напоминать другие гигантоклеточные опухоли [21–24]. Свое название «бурые» опухоли получили из-за появления коричневой окраски ткани в результате микрогеморрагий интерстиция и отложения гемосидерина. Встречаются «бурые» опухоли примерно у 2% пациентов с гиперпаратиреозом, такие образования обычно поражают нижнюю челюсть, ключицу, ребра и кости таза [25].

Остеобластокластомы, в отличие от «бурых» опухолей, представляют собой, как правило, доброкачественные остеогенные опухоли, а не очаги остеолизиса, однако по мере роста образования наблюдаются истончение и вздутие коркового слоя челюсти без его разрушения. При гистологическом исследовании выявляются многоядерные гигантские остеокласты, одноядерные гигантские остеобласты [26].

В описанном случае наблюдалось мультифокальное поражение костной ткани (переломы костей конечностей, компрессии тел позвонков), что в большей степени соответствовало «бурым» опухолям при тяжелом течении ПГПТ, а неоссифицирующей фиброме челюсти. По данным гистологического исследования препарата челюсти по месту жительства у пациентки изначально была диагностирована остеобластокластома. Однако при пересмотре препаратов в НМИЦ эндокринологии гистологически было подтверждено предположение о «бурой» опухоли. Это подчеркивает важность комплексной оценки результатов гистологического исследования в связи со схожестью микроскопических картин «бурых» опухолей и других опухолей челюсти.

С целью окончательного исключения синдрома HPT-JT данной пациентке выполнено секвенирование генов, ассоциированных с гиперпаратиреозом, мутации (в частности, в гене CDC73) выявлены не были.

Таким образом, представленный клинический случай является примером ретроспективной диагностики природы тяжелого гиперпаратиреоза у молодой женщины. В качестве итога анализа мы хотим суммировать имеющиеся на сегодняшний день практические рекомендации по дифференциальной диагностике тяжелого течения ПГПТ с объемным образованием челюсти.

## ЗАКЛЮЧЕНИЕ

Данный клинический случай демонстрирует значимость дифференциальной диагностики тяжелого течения ПГПТ с объемным образованием челюсти и важность оценки критериев трех состояний: тяжелого течения ПГПТ вследствие аденомы, рака ОЩЖ и синдрома гиперпаратиреоза с опухолью челюсти (HPT-JT).

## ДОПОЛНИТЕЛЬНАЯ ИНФОРМАЦИЯ

Источник финансирования. Работа выполнена по инициативе авторов без привлечения финансирования.

Конфликт интересов. Авторы декларируют отсутствие явных и потенциальных конфликтов интересов, связанных с публикацией настоящей статьи.

Участие авторов. Матюшкина А.С. — получение, анализ и интерпретация клинических данных, написание и редактирование статьи; Горбачева А.М. — получение, анализ и интерпретация клинических данных, написание и редактирование статьи; Еремкина А.К. — анализ клинических и литературных данных, написание и редактирование статьи; Ткачук А.В. — проведение гистологического и ИГХ-исследований; Мокрышева Н.Г. — анализ клинических данных, редактирование статьи. Все авторы одобрили финальную версию статьи перед публикацией, выразили согласие нести ответственность за все аспекты работы, подразумевающую надлежащее изучение и решение вопросов, связанных с точностью или добросовестностью любой части работы.

Согласие пациента. Пациент добровольно подписал информированное согласие на публикацию персональной медицинской информации в обезличенной форме в данном журнале.
